# Mechanisms of microRNA-mediated gene regulation in unicellular model alga *Chlamydomonas reinhardtii*

**DOI:** 10.1186/s13068-018-1249-y

**Published:** 2018-09-08

**Authors:** Sulin Lou, Ting Sun, Hui Li, Zhangli Hu

**Affiliations:** 1 Guangdong Key Laboratory of Plant Epigenetics, Guangdong Engineering Research Center for Marine Algal Biotechnology, Longhua Innovation Institute for Biotechnology, College of Life Sciences and Oceanography, Shenzhen University, Shenzhen, 518060 People’s Republic of China; 2 Key Laboratory of Optoeletronic Devices and Systems of Ministry of Education and Guangdong Province, College of Optoeletronic Engineering, Shenzhen University, Shenzhen, 518060 People’s Republic of China

**Keywords:** miRNA, Biogenesis, Mode of action, Regulatory mechanisms, Unicellular, Microalgae, *Chlamydomonas reinhardtii*

## Abstract

MicroRNAs are a class of endogenous non-coding RNAs that play a vital role in post-transcriptional gene regulation in eukaryotic cells. In plants and animals, miRNAs are implicated in diverse roles ranging from immunity against viral infections, developmental pathways, molecular pathology of cancer and regulation of protein expression. However, the role of miRNAs in the unicellular model green alga *Chlamydomonas reinhardtii* remains unclear. The mode of action of miRNA-induced gene silencing in *C. reinhardtii* is very similar to that of higher eukaryotes, in terms of the activation of the RNA-induced silencing complex and mRNA targeting. Certain studies indicate that destabilization of mRNAs and mRNA turnover could be the major possible functions of miRNAs in eukaryotic algae. Here, we summarize recent findings that have advanced our understanding of miRNA regulatory mechanisms in *C. reinhardtii*.

## Background

**Fig. 1:**
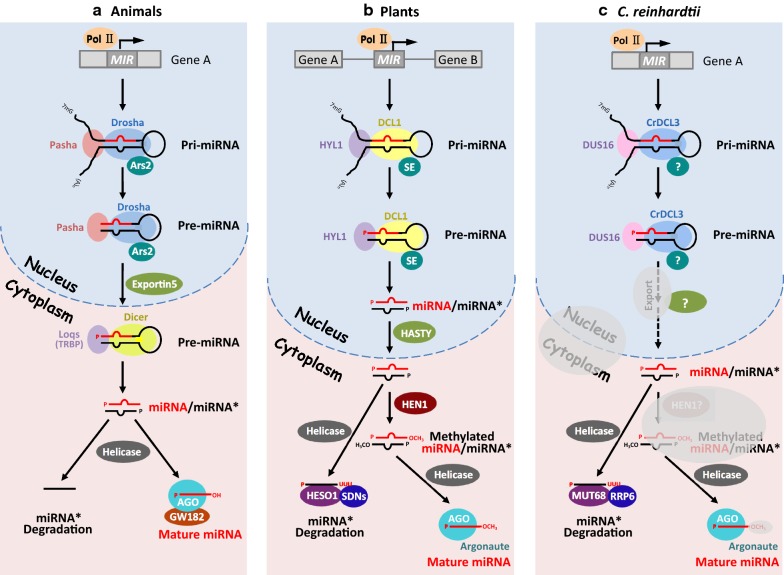
A schematic of canonical miRNA biogenesis in animals, plants and *C. reinhardtii*. Mature miRNAs are indicated in red, and miRNA* strands are in black. Homologs with similar functions are shown in the same color. Gray areas in the *C. reinhardtii* panel indicate unknown factors or processes. **a** In animals, miRNA genes (*MIR*) are embedded within the introns of protein-coding genes (Gene A, for example) and transcribed by RNA polymerase II (Pol II). Canonical animal miRNAs are processed by the nuclear RNase III enzyme Drosha, in cooperation with Pasha and Ars2. The precursor miRNA (pre-miRNA) is exported from the nucleus to the cytoplasm by Export5, and it is subsequently cut into a miRNA/miRNA* duplex with 2 nt 3′ overhangs by Dicer, acting together with Loqs. One strand of the duplex is degraded, and the other mature miRNA is loaded onto RISC, whose core component is an AGO protein. **b** In plants, *MIR* genes are embedded within the noncoding sequences between protein-coding genes (Gene A and Gene B, for example) and are also transcribed by Pol II. Canonical plant miRNAs are produced by the nuclear RNase III Dicer-like1 (DCL1), assisted by HYL1/DRB1 and SERRATE (SE). DCL1 is responsible for both steps of miRNA processing to produce the miRNA/miRNA^*^ duplex, which is then transported to the cytoplasm by HASTY. The miRNA/miRNA^*^ duplex undergoes 2′-*O*-methylation modification by HEN1 at the 3′ end. One strand of the methylated duplex is degraded, and the other mature miRNA is loaded onto RISC, whose core component is an AGO protein. Plant miRNAs that are not methylated are subject to 3′ end uridylation by the poly(U) polymerase HESO1, resulting in subsequent degradation. **c** In *Chlamydomonas*, *MIR* genes are embedded within the introns of protein-coding genes (Gene A, for example) and transcribed by Pol II, similar to animal *MIR*. *Chlamydomonas* miRNAs are processed by the nuclear RNase III enzyme *Cr*DCL3, in cooperation with DUS16. MUT68 is involved in 3′ end uridylation of miRNAs and may mediate miRNA degradation in cooperation with RRP6. The export protein responsible for transporting *Chlamydomonas* miRNAs from the nucleus to the cytoplasm is unknown. Whether mature miRNAs in *C. reinhardtii* are methylated is also presently unknown (gray areas in the diagram)

MicroRNAs (miRNAs) are endogenous non-coding RNAs approximately 21–24 nucleotides (nt) in length with significant roles in post-transcriptional gene regulation. MiRNAs were first identified in 1993 in *Caenorhabditis elegans* and subsequently in *Arabidopsis thaliana*, *Drosophila*, mouse, human and other multicellular organisms [1–7]. MiRNAs were not described in unicellular *Chlamydomonas reinhardtii* until 2007, much later than the initial reports in animals and plants [8, 9]. In animals, mature miRNAs are embedded in the duplexed stem loop structures of primary miRNAs (pri-miRNAs) and are excised by the ribonuclease III (RNase III) enzyme Drosha [10, 11] accompanied by the double-stranded RNA-binding domain (dsRBD) protein Pasha and Ars2 [12, 13]. The precursor miRNA (pre-miRNA) is subsequently exported from the nucleus to the cytoplasm by Exportin 5 [14, 15] and processed into a miRNA/miRNA* duplex by another RNase III enzyme, Dicer. One strand of the duplex is the mature miRNA, which is loaded onto the RNA-induced silencing complex (RISC) [16], whose core component is an Argonaute protein (AGO) [17] (Fig. 1a). Although plant miRNA biogenesis also involves two cleavage steps, it differs in some important ways from the process in animals (Fig. 1a). First, the two precise cleavage steps of plant miRNA biogenesis are executed in the nucleus by the same RNase III enzyme, Dicer-like 1 (DCL1), with the assistance of HYL1 and SE [18–26]. Another difference is that plant miRNAs are typically 21 nt in length, while animal miRNAs are longer, generally 22–23 nt in length. However, the critical difference is that after the two cleavage steps, plant miRNAs are modified by HEN1 methylation at the 3′ end, which protects them from degradation [27–32] (Fig. 1b). In contrast, the modification by HEN1 is uncommon for animal miRNAs. To date, *Cr*DCL3, assisted by DUS16, is the only RNase III enzyme known to cleave pri-miRNAs to generate miRNA duplexes in unicellular *C. reinhardtii* [33–35]. Whether the cleavage of *Chlamydomonas* miRNAs occurs in the nucleus or in the cytoplasm is still unknown (Fig. 1c).

Mature miRNAs are loaded onto AGOs to activate RISCs, which then cleave the target mRNA or prevent translation through the complementarity of miRNAs to their target mRNAs [17, 36–38]. Plant miRNAs require near-perfect complementarity to direct the cleavage of target mRNAs [2, 3, 39]. In contrast, animal miRNAs only require complementarity of the seed sequence and hence a broader range of targets, with one animal miRNA targeting several mRNAs. Animal miRNAs usually inhibit the translation of target proteins [40, 41]. As one example in *Chlamydomonas*, complementary pairing with the seed sequence of the miRNA cre-miR1174.2 was found to be sufficient to trigger repression of target transcripts [42]. Nevertheless, it remains unclear whether the mode of action of miRNA in *Chlamydomonas* is more similar to those of animal miRNAs or plant miRNAs. Key factors involved in miRNA biogenesis and regulation identified in animals and higher plants are listed in Table 1. The corresponding factors and processes in unicellular *C. reinhardtii* are not as well understood, but in recent years, some progress has been made towards characterizing miRNA regulatory mechanisms in this species.

**Table 1:** List of key miRNA pathway genes in animals, plants and algae

	Gene (gene products)	Function in miRNA pathway	Animals (*C. elegans*)	Plants (*A. thaliana*)	Algae (*C. reinhardtii*)
miRNA biogenesis	Dicer (Ribonuclease III)	Cleavage of pre-miRNA or pri-miRNA	Drosha [11, 12]; Dicer1 [81, 82]	DCL1 [18, 19, 21]	CrDCL3 [33]
	DRB (Double-stranded RNA binding Protein)	Assist efficient and precise cleavage of pri-miRNA through interaction with Dicer	Pasha [12, 13]	DRB1(HYL1) [20, 22, 23, 26]; DRB2 [87–89]; DRB5 [90]	DUS16 [35, 91] ?
	Others	Other nuclear regulators in primary microRNA processing	Ars2 [75, 76]	SE [24, 25]	?
miRNA export	Exportin	Export pre-miRNA or miRNA/miRNA* from nucleus to cytoplasm	Exportin5 [14, 15]	HASTY [79, 80]	?
miRNA action	AGO (Argonaute)	Central component of RISC, mediate miRNA-directed regulation of endogenous gene expression	AGO1 [17]; AGO2 [34]	AGO1 [54, 55]; AGO2 [56]; AGO4 [57]; AGO5 [36]; AGO7 [58]; AGO10 [107]	CrAGO3 [34, 74]
	RISC (RNA-induced silencing complex)	This complex consists of several proteins and RNA molecules that altogether trigger transcript degradation or preventing translation of target mRNA	eIF6 [59, 60]; eIF2C2, Gemin4, Gemin3 [61–63]	HSP90 [107]; EMA1 [84]; TRN1 [84]	?
mature miRNA modification	HEN1 (small RNA methyl transferase)	Methylation prevents miRNAs from degradation triggered by uridylation	–	HEN1 [18, 27–29]	?
miRNA degradation	Uridylation	Terminal nucleotidyl transferase that prefers to add untem-plated uridine to the 3′ end of RNA	–	HESO1 [108, 109]; URT1 [110]; SDN1, SDN2 [30, 107–109]	MUT68 [118]; RRP6 [118]

Unidentified genes in *C. reinhardtii* are indicated with ‘?’

Artificial miRNA (amiRNA) technology was developed to knock down the expression of specific target genes. Generally, an endogenous miRNA precursor is used as the backbone to construct a highly specific and high-throughput amiRNA system [43–48]. Short tandem target mimic (STTM) technology is another approach used in plants and animals to suppress the functions of specific miRNAs [49–53]. In *C. reinhardtii*, however, there have not been any reports in which STTM technology was successfully applied.

In this brief review, we summarize findings from previous studies about miRNA biogenesis and modes of action in plants, animals and unicellular *C. reinhardtii*, including our own research findings, to shed light on the evolutionary divergence of miRNA regulatory mechanisms between multicellular and unicellular organisms.

## Discovery and biogenesis of miRNAs

### Large-scale discovery of miRNAs

In the first report of miRNAs, the heterochronic gene *lin*-*4* in *C. elegans* was found not to encode a protein but a small ~ 22 nt transcript complementary to the 3′ untranslated region (UTR) of *lin*-*14* mRNA that temporally decreases LIN-14 protein levels in the first larval stage (L1) [64, 65]. Thereafter, the *C. elegans* heterochronic switch gene *let*-*7* was found to encode a temporally regulated 21 nt RNA complementary to sites in the 3′UTR regions of *lin*-*14*, *lin*-*28*, *lin*-*41*, *lin*-*42* and *daf*-*12*, thereby regulating the developmental timing of *C. elegans* [66]. At that time, these short RNAs were termed small temporal RNA (stRNA), which is the predecessor of the term miRNA.

MiRNAs are key players in post-transcriptional gene regulation that target mRNAs, and they have been reported in various multicellular animals and plants as well as viruses [67, 68]. In 2001, three groups published their findings in the same issue of *Science*, reporting the prediction of precursor structures of *Drosophila melanogaster* (*D. melanogaster*) miRNAs and human miRNAs [69], the identification of 55 *C. elegans* miRNAs [70] and 15 new *C. elegans* miRNAs through a combination of cloning and bioinformatic approaches [10].

MiRNAs in the dicot *A. thaliana* were first isolated in 2002, followed by the functional characterization of many key miRNA metabolism factors. For example, DICER-LIKE 1 (DCL1) and HUA ENHANCER 1 (HEN1) are required for miRNA accumulation in *A. thaliana* [1, 3, 18]. In the monocot rice, some new miRNAs evolved independently, based on comparison with *Arabidopsis* [7]. In bryophytes, the oldest land plants, miRNAs have also been identified and are considered to play important regulatory roles [5]. The wide distribution of miRNAs and diversity of species hosting this RNA regulatory mechanism suggest that miRNAs have extensive functions in eukaryotic cells.

MiRNAs were once thought to exist only in higher multicellular eukaryotes as a gene expression regulatory system to increase biocomplexity. In 2007, miRNAs were reported in lower unicellular green alga *C. reinhardtii* by two independent research groups using large-scale high-throughout sequencing analysis [8, 9]. They showed that miRNAs in *C. reinhardtii* could cut target mRNAs in vivo and in vitro and also found that the presumptive miRNA precursors were similar to those of higher plants. Further research showed that the expression of some miRNAs (or candidate miRNAs) increased or decreased during *C. reinhardtii* gametogenesis, indicating spatiotemporal specificity of these miRNAs. Additionally, there were other kinds of sRNAs reported, including small interfering RNAs (siRNAs) similar to *trans*-acting siRNAs in plants and some siRNAs derived from protein-coding genes and transposons [9]. The common features of miRNAs between unicellular algae and higher plants indicate that the complex miRNA post-transcriptional regulatory system evolved earlier than multicellular organisms and may be an ancient regulatory mechanism [8, 9].

Many new miRNAs have been characterized through stress treatment of *C. reinhardtii*. We predicted 85 known miRNAs and 225 novel miRNAs from sulfur-deprived *C. reinhardtii*; among these miRNAs, 47 were differentially expressed (mostly upregulated) and 13 were specific to the sulfur-deprived condition [71, 72], along with genome-wide long non-coding RNA were screened, identified and characterized [73]. These findings strongly suggested that sulfur deprivation significantly influences miRNA expression patterns.

Many other miRNAs in *C. reinhardtii* have been identified based on their association with key proteins. For example, AGO3-associated miRNAs were identified by affinity purification in *C. reinhardtii*, which yielded 45 unique miRNAs including 32 that were previously unknown [74]. Mutants in the *DICER*-*LIKE 3* (*DCL3*) gene failed to produce miRNAs, indicating that most miRNAs are produced by DCL3-mediated cleavage in *C. reinhardtii* [33]. Recently, pri-miRNAs associated with the RNA-binding protein Dull slicer 16 (DUS16) were characterized in *C. reinhardtii* by sequencing the sRNAs of the *dus16* mutant; 35 pri-miRNA genes were predicted de novo, 9 of which were novel [35]. The high degree of overlap between DUS16-dependant pri-miRNAs and DCL3-dependant pri-miRNAs indicates a cooperative relationship between DUS16 and DCL3 in *C. reinhardtii* pri-miRNA processing.

### MiRNA biogenesis

MiRNA biogenesis involves two steps in both animals and plants. In animals, the long, capped and polyadenylated pri-miRNAs are synthesized by RNA polymerase II then cut by the RNAse III enzyme Drosha, acting with double-stranded RNA-binding (DRB) protein Pasha and Ars2 [75, 76], creating a ~ 70 nt stem loop known as a precursor miRNA (pre-miRNA). The pre-miRNA is exported from the nucleus to the cytoplasm by Exportin 5, and then cut into a miRNA/miRNA* duplex with 2 nt 3′ overhangs by Dicer, another RNAse III enzyme. The dsRBD proteins are also involved in this process, such as Loquacious (Loqs) in *Drosophila* and the trans-activator RNA (tar)-binding protein (TRBP) in humans [77]. One strand of the duplex is degraded, and the other single-stranded mature miRNA is loaded onto RISC, whose core component is an AGO protein [78] (Fig. 1). In plants, the Dicer homolog DCL1, assisted by the RNA-binding proteins HYPONASTIC LEAVES1 (HYL1/DRB1) and SERRATE (SE), is responsible for both steps of miRNA processing to produce the miRNA/miRNA* duplex in the nucleus. The duplex is subsequently transported to the cytoplasm by HASTY [79, 80] (Fig. 1).

There are notable differences in miRNA biogenesis between plants and animals. First, the locations of miRNA-encoding genes differ. In plants, the majority of these genes are located in intergenic regions between protein-coding genes and have independent transcription elements. In animals, most miRNA-encoding genes reside in the introns of protein-coding genes. Second, plant miRNAs are synthesized entirely within the nucleus, with the two cleavage steps performed by the RNAse III enzyme DCL1, which is endonuclear. The first cleavage of animal miRNA processing takes place in the nucleus, while the second cleavage occurs in the cytoplasm [81, 82]. Third, miRNA length differs between plants and animals. Plant miRNAs are typically 21 nt, while animal miRNAs are a little longer at 22–24 nt, which may reflect the different enzymes responsible for the first processing cleavage (DCL1 in plants and Drosha in animals) (Fig. 1). Finally, most plant miRNAs are methylated at the 3′ end to protect them from degradation after the two cleavage steps, whereas few animal miRNAs exhibit such modifications.

A recent study in *C. reinhardtii* found that roughly 50% of *Chlamydomonas* miRNA genes are embedded within the introns of protein-coding genes, which is similar to the finding in animals that approximately 30% of miRNA genes are located in the introns of protein-coding genes [33, 41, 83]. In contrast, few cases of intronic miRNA genes are known in plants; instead, plant miRNA genes typically occur in intergenic regions. Among three DCL paralogs, DCL3 of *C. reinhardtii* (*Cr*DCL3) is in charge of cleaving pri-miRNAs into mature miRNAs, functioning similarly to Drosha and Dicer in animals and DCLs in plants. Interestingly, *Cr*DCL3 lacks a PAZ domain but has a proline-rich region, which is similar to Drosha in animals (Fig. 1) [33, 84]. In unicellular *C. reinhardtii*, miRNA biogenesis is poorly understood but seems to be more similar to the process in metazoans than in land plants.

As with the essential partners that interact with the nucleases DCL and Drosha in multicellular organisms, a DRB protein is similarly involved in the precise processing of pri-miRNAs in *C. reinhardtii*. In *A. thaliana*, HYL1/DRB1 and DRB2 cooperate with DCL1 to ensure efficient and precise cleavage of pri-miRNAs [20, 22, 23, 85–90], with HYL1/DRB1 directing guide strand selection from the miRNA duplex [26]. To date, only one DRB protein, DUS16, has been implicated in pri-miRNA processing in *C. reinhardtii*. DUS16, as a component of a microprocessor complex, interacts with *Cr*DCL3 and recognizes pri-miRNA transcripts cotranscriptionally [91], which is similar to the functions of DRB proteins involved in miRNA biogenesis in animals and land plants (Fig. 1). In our research, we have isolated and identified several other DRBs whose loss leads to reduced miRNA abundance, especially miRNA B (miR B) and miRNA C (miR C) (data not yet published).

No miRNAs appear to be conserved between *Chlamydomonas* and land plants. Moreover, only one miRNA is homologous between *Chlamydomonas* and *Volvox carteri*, another green alga [41, 92–96]. This suggests a high divergence rate for miRNAs between microalgae and land plants, and even between different green algal lineages. Thus, changes in the miRNA pathway from unicellular to multicellular organisms appear to reflect a complex and rapid evolution.

## Modification of miRNAs

### Methylation of miRNAs

In contrast to animal miRNAs, mature miRNAs in plants undergo 2′-*O*-methylation modification by HEN1 at the 3′ end, which helps to maintain their stability and helps prevent exonucleolytic degradation. *HEN1* was initially identified as a floral pattering gene with multiple roles in floral organ identity specification. In *Arabidopsis hen1* mutants, miRNA abundance was found to be largely reduced, while miRNA size increased, with one–five U residues added to the 3′ ends of miRNAs. In vitro, HEN1 recognizes and acts on miRNA/miRNA* duplexes but not on pre-miRNAs or single-stranded miRNA or miRNA* [18, 27–29, 31, 97, 98]. Further research showed that this methylation modification at the 3′ end also occurs on plant siRNAs and animal Piwi-interacting RNAs (piRNAs) [99–104].

The *C. reinhardtii* homolog of HEN1 is poorly characterized, and it is unknown whether *Chlamydomonas* miRNAs require methylation by HEN1. We have cloned the *HEN1* gene of *C. reinhardtii* and predicted and analyzed its protein structure, which harbors a methyltransferase domain. We also observed that miRNA abundance in the *Chlamydomonas hen1* mutant is generally reduced (data not yet published).

### Uridylation of miRNAs

Plant miRNAs that are not methylated are subject to 3′ end uridylation by a poly(U) polymerase, resulting in subsequent degradation. In *C. elegans*, the 5′-to-3′ exoribonuclease XRN2 is responsible for the degradation of mature miRNAs [105]. Recent studies have associated 3′ truncation and 3′ uridylation with miRNA degradation [99, 103, 106, 107].

Nucleotidyl transferases, such as HEN1 suppressor 1 (HESO1), play a crucial role in miRNA uridylation in *Arabidopsis*; specifically, these enzymes may lead to the uridylation of AGO1-bound unmethylated miRNAs to trigger their degradation [108–111]. The 3′-to-5′ exoribonuclease SMALL RNA DEGRADING NUCLEASE (SDN) family is also implicated in the turnover and degradation of miRNAs in *A. thaliana*. A recent study showed the involvement of two members of the SDN family, SDN1 and SDN2, in miRNA 3′ truncation; the 3′ truncated miRNAs were subsequently tailed by HESO1, leading to their degradation in *Arabidopsis*. In addition, these SDNs were also required for the degradation of AGO10-bound miR165/6 [107, 109, 112].

In *C. elegans* and mammalian cells, miRNA let-7 regulates lin-28, whereas lin-28 binds to the let-7 precursor in the cytoplasm and stimulates 3′ tail uridylation by a poly(U) polymerase, resulting in precursor degradation and reduced let-7 miRNA [113–116]. Nonetheless, miRNA uridylation does not always lead to degradation. For instance, uridylation of mature miR-26 reduces its activity in terms of target regulation without affecting its abundance in mammalian cells [115].

In *Chlamydomonas*, miRNA degradation is not well understood, but the RNAi defective mutant Mut-68, lacking the nucleotidyltransferase MUT68, has shed some light on the degradation of miRNAs and siRNAs [117] MUT68 is involved in 3′ end uridylation of miRNAs in vivo and may mediate miRNA degradation in cooperation with RRP6, a subunit of the exosome (a 3′-to-5′ exonuclease); depleting RRP6 also increases miRNA and siRNA accumulation in vivo. MUT68 carries out the degradation of longer mRNAs generated by RISC cleavage, and it may preferentially uridylate small RNAs and adenylate RISC-cleaved transcripts. Ultimately, degradation is a quality control mechanism for the removal of dysfunctional or damaged small RNAs [118, 119], and the processes of miRNA degradation and biogenesis must be balanced for proper regulatory function in cells.

## Mode of action and utilization of miRNAs

### MiRNA mode of action in *C. reinhardtii*

**Fig. 2:**
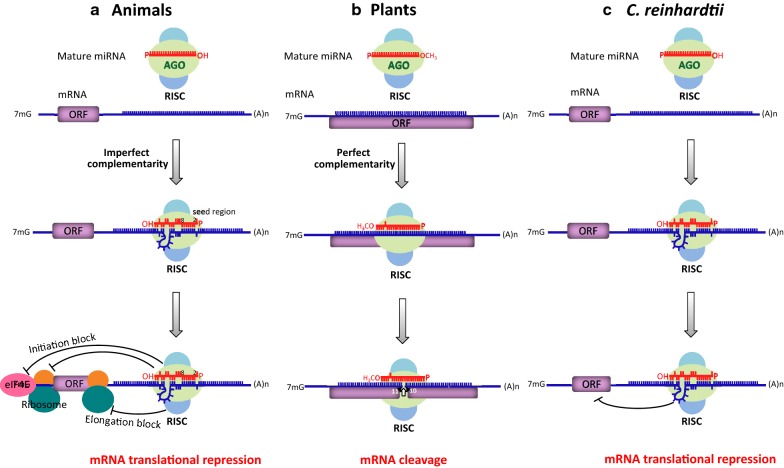
MiRNA modes of action in animals, plants and *C. reinhardtii*. Mature miRNAs are indicated in red, and mRNAs are in blue. The open reading frames of genes are represented with purple boxes. **a** In animals, mature miRNAs bind to the 3′UTR of their target mRNAs to trigger translation repression. The complementarity of animal miRNAs to their target mRNAs is imperfect: only the seed sequence (positions 2–8 of the miRNA) needs to be perfectly paired. Animal miRNAs often induce translational repression of targets by blocking translation initiation or elongation. **b** In plants, mature miRNAs bind to the coding region of their target mRNAs. Plant miRNAs typically exhibit near-perfect complementarity to their target mRNAs, leading to endonucleolytic slicing between positions 10 and 11 of miRNA/mRNA hybrids and subsequent degradation of the target mRNA. **c** In *Chlamydomonas*, mature miRNAs prefer to bind to the 3′UTR of their target mRNAs to trigger translation repression, similar to animal miRNAs. The complementarity of *Chlamydomonas* miRNAs to their target mRNAs is imperfect; perfect base pairing of the seed sequence is sufficient to induce moderate repression of target mRNAs

Mature miRNAs are loaded onto AGO-containing RISC, which performs cleavage and/or translation repression of target mRNAs through the complementarity of miRNAs to their target mRNAs. Plant miRNAs often exhibit almost perfect complementarity to their target mRNAs, leading to endonucleolytic slicing between positions 10 and 11 of miRNA/mRNA hybrids and subsequent degradation of the target mRNA (Fig. 2) [2, 4, 6, 39, 41, 42, 120, 121]. The complementarity of animal miRNAs to their target mRNAs is usually not as strict: only the seed sequence (positions 2–8 of the miRNA) needs to be perfectly paired, with imperfect hybridization permissible at positions 9–12 of the miRNA central region (Fig. 2) [6, 120]. Due to the loose sequence complementarity, a given animal miRNA is often predicted to have multiple target mRNAs, whereas a given plant miRNA would have far fewer targets. In terms of mode of action, animal miRNAs often induce translational repression of targets by blocking translation initiation or elongation or by deadenylation (Fig. 2), resulting in a relatively weaker modulatory effect of target mRNA and protein levels compared to cleavage [121–125]. Cleavage and translation repression are the common modes of action in plants and animals, respectively, exceptions abound, including miRNA-induced translation inhibition in plants and mRNA cleavage in animals [39, 126, 127]. MiRNA-mediated translation inhibition in plants also requires perfect complementarity and depends on the GW-repeat protein SUO, which is not homologous to the animal counterpart GW182 [41, 128, 129]. These findings suggest that a given miRNA may simultaneously carry out two modes of action at the same time, with one mode being dominant.

MiRNA regulatory mechanisms remain poorly understood in unicellular *C. reinhardtii* compared to multicellular eukaryotes. A mismatch tolerance assay of *Chlamydomonas* miRNA cre-miR1174.2 showed that complementary pairing of the 5′ end seed sequence was sufficient to trigger moderate repression of the target transcript, while pairing of the 3′ end sequence between the miRNA and its target was not significant for post-transcriptional regulation [42]. These features of *Chlamydomonas* miRNA target recognition differ from the near perfect complementarity required for miRNAs in higher plants but are similar to the characteristics of metazoan miRNAs (Fig. 2) [33, 130]. Thus, a given miRNA may have more than one target mRNA in *Chlamydomonas*, which would increase the difficulty of targeting a specific gene. MiRNA-mediated target mRNA cleavage also exists in *Chlamydomonas* [131].

In *Chlamydomonas*, there are three AGO paralogs (AGO1, AGO2 and AGO3), and AGO3 is critical for miRNA-mediated target transcript cleavage and translational repression. In contrast, AGO2 appears to have a limited contribution to gene silencing, and AGO1 is potentially uninvolved in miRNA-mediated post-transcriptional activity [34]. Another difference appears to exist between the miRNA-mediated RNA silencing mechanisms of *Chlamydomonas* and plants. Transcriptome analysis revealed more transcripts in the *dcl3* mutant compared to wild-type, reflecting the role of DCL3 in cutting miRNA precursor structures embedded in mRNAs but not the cleavage of target mRNA [33, 94]. *C. reinhardtii* miRNAs bind to the 3′UTR of their target mRNAs to trigger translation repression. Moreover, this miRNA-mediated inhibition occurs at a post-initiation step of protein synthesis, which may affect the function or structural conformation of translating ribosomes [42, 132].

## Artificial miRNAs and STTM in *C. reinhardtii*

### Artificial miRNAs

Following the large-scale identification of *Chlamydomonas* miRNAs, artificial miRNA (amiRNA) systems were developed for knockdown in *Chlamydomonas* as a reverse genetics approach. For example, amiRNAs targeting the *MAA7* and *RBCS1/2* genes were constructed, using an endogenous *Chlamydomonas* miRNA precursor as the backbone that could cleave and decrease the expression of their respective target transcripts [44]. Based on the ligation of DNA oligonucleotides, a highly specific and high-throughput amiRNA system was developed for targeted gene silencing in *Chlamydomonas* [43]. In light of possible off-target effects, an inducible amiRNA system was developed thereafter, incorporating the *NIT1* promoter, which is repressed by ammonium and activated by nitrate; heat shock factor 1 (HSF1) was the designated target, and the analysis demonstrated that HSF1 is a crucial thermotolerance regulator in *Chlamydomonas* [45]. In a study using amiRNA technology to target pyruvate formate lyase (PFL1), which catalyzes the conversion of pyruvate to acetyl-CoA and formate under anoxic conditions, PFL1 protein and mRNA levels were both decreased by 80–90% in two transformants [46]. An optimized one-step amiRNA precursor construction was also established for high-throughput assays, with potential utility not only in *Chlamydomonas* but also in other model organisms. Specifically, the *Gaussia princeps* luciferase (G-Luc) gene was positioned between the promoter and the amiRNA precursor to facilitate screening and detection, and the inducible promoter of the nitrate reductase gene was added to the amiRNA precursor vector construct [47].

Our previous research shows using an amiRNA strategy to knock down a photosystem II-related protein, oxygen evolving enhancer (OEE2), higher H_2_ production was observed in *Chlamydomonas* [48]. Afterwards, a blue light-inducible system was constructed to optogenetically regulate an amiRNA (amiR-D1) and, consequently, regulation of its target gene. Hydrogen production was enhanced in *Chlamydomonas* using this system [133, 134]. In addition, using amiRNA to inhibit the phosphoenolpyruvate carboxylase increased fatty acid production in a green microalga *Chlamydomonas* [133]. These particular examples have important economic potential in terms of providing sustained H_2_ and fatty acid productions by green algae for industrial applications, avoiding some of the inconvenience of traditional hydrogen production methods.

### STTM in *C. reinhardtii*

In recent years, short tandem target mimic (STTM) technology has been shown to be an effective approach for blocking the functions of specific miRNAs and siRNAs in both plants and animals [49–52, 135–137]. However, in our research, we have observed non-specific effects when using STTM to knock down miRNAs in *C. reinhardtii* (data not yet published). There are several possible explanations for the differences in STTM efficacy between unicellular *C. reinhardtii* and multicellular plants and animals. For instance, the cellular components and the degradation mechanisms may be distinct. The optimal “spacer” length for STTM may also differ. More broadly, these differences suggest a disparate evolution rate of the miRNA pathway among plants, animals and *C. reinhardtii*.

## Conclusions and future perspective

MiRNAs, as a class of endogenous non-coding RNAs, play an essential role in post-transcriptional gene regulation in plants and animals. Recent studies have helped to establish that miRNAs may also have an important role in unicellular microalgae. Some encouraging results have been reported, for instance, endogenous miRNA showed promising regulatory roles on H_2_ production in *C. reinhardtii* [138]. In addition, by artificial miRNAs we achieved successfully to regulate the productions of H_2_, fatty acid and carotenoids [133, 134, 139–143]. Understanding the mechanism of action of miRNAs in *C. reinhardtii* is of great significance to further utilize microalgae as biofuel- or bioproduct-based resources. Unlike the relatively clear mechanisms of miRNA-mediated gene regulation in plants and animals, however, the corresponding processes in *C. reinhardtii* remain vague. From the presently available research, we know there are important similarities and differences in the miRNA mechanisms of unicellular *C. reinhardtii* and multicellular organisms. MiRNA-encoding genes are found within the introns of protein-coding genes in *C. reinhardtii*, similar to miRNA genes in animals. Protein structure analysis of *Chlamydomonas* DCL3 showed that it resembles animal Drosha, which lacks a PAZ domain. Additionally, *Chlamydomonas* miRNAs usually bind to the 3′UTR of their targets, with seed sequence base pairing being sufficient for targeting. The mainstream academic view is that miRNA biogenesis and regulation in *C. reinhardtii* are more similar to that in animals than that in plants.

Research on the miRNA pathway in unicellular *C. reinhardtii* faces particular challenges. Compared with *Arabidopsis* and animals, the bigger gene size and higher GC content of key genes in the *Chlamydomonas* miRNA pathway make it more difficult to characterize their functions. The export protein responsible for transporting *Chlamydomonas* miRNAs from the nucleus to the cytoplasm is presently unknown. Is Dus16 the only DRB protein acting with *Cr*DCL3 in *Chlamydomonas* miRNA biogenesis, or are there other DRBs in *Chlamydomonas* with different roles that affect the miRNA mode of action, as in *Arabidopsis*?

The localization of the core miRNA biogenesis protein *Cr*DCL3 in *C. reinhardtii* is also unknown. Does miRNA processing and *Cr*DCL3 activity occur in both the nucleus and cytoplasm, or is *Cr*DCL3 responsible for cleavage only in the nucleus? Are mature miRNAs in *C. reinhardtii* methylated by HEN1? These remain open questions in *Chlamydomonas* (represented by gray areas in Fig. 1) to be addressed by future research. Assessing the miRNA pathway functions of *C. reinhardtii* proteins homologous to known miRNA-related proteins will help to improve our understanding of the evolution of the miRNA pathway in eukaryotes.

As a unicellular organism, *C. reinhardtii* has no tissue and organ differentiation and thus lacks apparent and easily observable phenotypes. In our studies, several loss-of-function mutants affecting critical miRNA pathway genes exhibit regular growth, whereas loss of function of these key genes in plants or animals always results in abnormal growth. Does this mean that miRNAs in unicellular *C. reinhardtii* are not as important as we expected? Future research regarding miRNA production and regulation in the model green alga *C. reinhardtii* should address this question and shed light on the evolution of miRNA mechanisms.

With the rapid improvement and reduced cost of sequencing technology, miRNAs have been identified in more and more eukaryotic organisms. Although the mechanism of action of miRNA has been only studied in model microalga *C. reinhardtii* at present, many other algae, such as *Volvox carteri*, *Phaeodactylum tricornutum*, *Porphyridium purpureum* and brown algae have been successively identified for miRNAs [95, 144–146]. In this regard, miRNA sequencing and annotation in other species of unicellular microalgae, combined with analysis of miRNA mode of action (cleavage of targets or translation inhibition) in future, will shed light on open questions about miRNA evolutionary turnover.

## Abbreviations

amiRNA: artificial miRNA
*A. thaliana*: *Arabidopsis thaliana*
AGO: Argonaute
*C. elegans*: *Caenorhabditis elegans*
*C. reinhardtii*: *Chlamydomonas reinhardtii*
dsRBD: double-stranded RNA-binding domain
DCL1: DICER-LIKE 1
DCL3: DICER-LIKE 3
*Cr*DCL3: DCL3 of *C. reinhardtii*
*D. melanogaster*: *Drosophila melanogaster*
DUS16: Dull slicer 16
G-Luc: *Gaussia princeps* luciferase
HSF1: heat shock factor 1
HESO1: HEN1 suppressor 1
HEN1: HUA ENHANCER 1
HYL1/DRB1: HYPONASTIC LEAVES1
Loqs: Loquacious
miRNAs: microRNAs
miR B: miRNA B
miR C: miRNA C
OEE2: oxygen evolving enhancer
piRNAs: Piwi-interacting RNAs
pre-miRNA: precursor miRNA
pri-miRNAs: primary miRNAs
PFL1: pyruvate formate lyase
RNase III: ribonuclease III
RISC: RNA-induced silencing complex
SE: SERRATE
STTM: short tandem target mimic
siRNAs: small interfering RNAs
stRNA: small temporal RNA
SDN: SMALL RNA DEGRADING NUCLEASE
TRBP: trans-activator RNA (tar)-binding protein
UTR: untranslated region
